# A modified endoscopic submucosal dissection for a superficial hypopharyngeal cancer: a case report and technical discussion

**DOI:** 10.1186/s12885-017-3685-7

**Published:** 2017-11-06

**Authors:** Lianjun Di, Kuang-I Fu, Rui Xie, Xinglong Wu, Youfeng Li, Huichao Wu, Biguang Tuo

**Affiliations:** 1 Department of Gastroenterology, Affiliated Hospital, Zunyi Medical College, Zunyi, 563003 China; 2 Department of Endoscopy, Kanma Memorial Hospital, Tokyo, Japan; 3 Department of pathology, Affiliated Hospital Zunyi Medical College, Zunyi, China

**Keywords:** Hypopharyngeal cancer, Case report, ESD, Transparent hood

## Abstract

**Background:**

Adequate working space and a clear view for the dissected lesion are crucial for endoscopic submucosal dissection (ESD). Pharyngeal ESD requires that an otorhinolaryngologist creates working space by lifting the larynx with a curved laryngoscope. However, many countries do not have this kind of curved laryngoscope, and the devices could interfere with endoscope because of the narrow space of the pharynx. To overcome these issues, we used a transparent hood (Elastic Touch, slit and hole type, M (long), Top company, Tokyo Japan) instead of the curved laryngoscope to create adequate working space by pushing the larynx, and pharyngeal ESD could be done by gastroenterologists.

**Case presentation:**

A 64-year-old male patient was admitted to our hospital because of chronic persistent swallowing dysfunction for 2 years. Oesophagogastroduodenoscopy showed a superficial hypopharyngeal cancer in the right pyriform sinus. We used a transparent hood (Elastic Touch, slit and hole type, M (long), Top company, Tokyo Japan) instead of the curved laryngoscope to create adequate working space by pushing the larynx, and dental floss tied to a haemoclip was applied to create counter traction during ESD. The lesion was pathologically confirmed as superficial squamous cell carcinoma and resected completely.

**Conclusions:**

This is the first report of modified ESD for a superficial hypopharyngeal cancer. The modified ESD enables early pharyngeal superficial cancer to be removed completely under endoscope by gastroenterologist.

**Electronic supplementary material:**

The online version of this article (10.1186/s12885-017-3685-7) contains supplementary material, which is available to authorized users.

## Background

Endoscopic submucosal dissection (ESD) is an effective procedure for the treatment of superficial mesopharyngeal and hypopharyngeal cancers [1]. The studies from Muto et al. [2] and Satake et al. [3] showed that the disease-specific survival and 5-year overall survival were from 97% to 100% and 71% to 85%, respectively, after transoral endoscopic treatment. Endoscopic treatment is less invasive and preserves swallowing and speech functions in comparison with traditional surgical approaches and radiotherapy. However, ESD of the pharyngeal region has not been widely used still because of the limitation of the device manoeuvrability and the complex structure of the region, and because conventional ESD requires an otorhinolaryngologist to create adequate working space by lifting the larynx with a curved laryngoscope, which takes time and increases medical expenses. Another difficulty for the procedure is that the narrow space of the pharynx makes endoscope and other devices to interfere with each other. To overcome these issues, we used a transparent hood (Elastic Touch, slit and hole type, M (long), Top company, Tokyo Japan) instead of the laryngoscope to provide adequate working space and used dental floss tied to a haemoclip to provide a well-visualized dissecting line during ESD of superficial cancer in the hypopharynx region.

## Case presentation

**Fig. 1:**
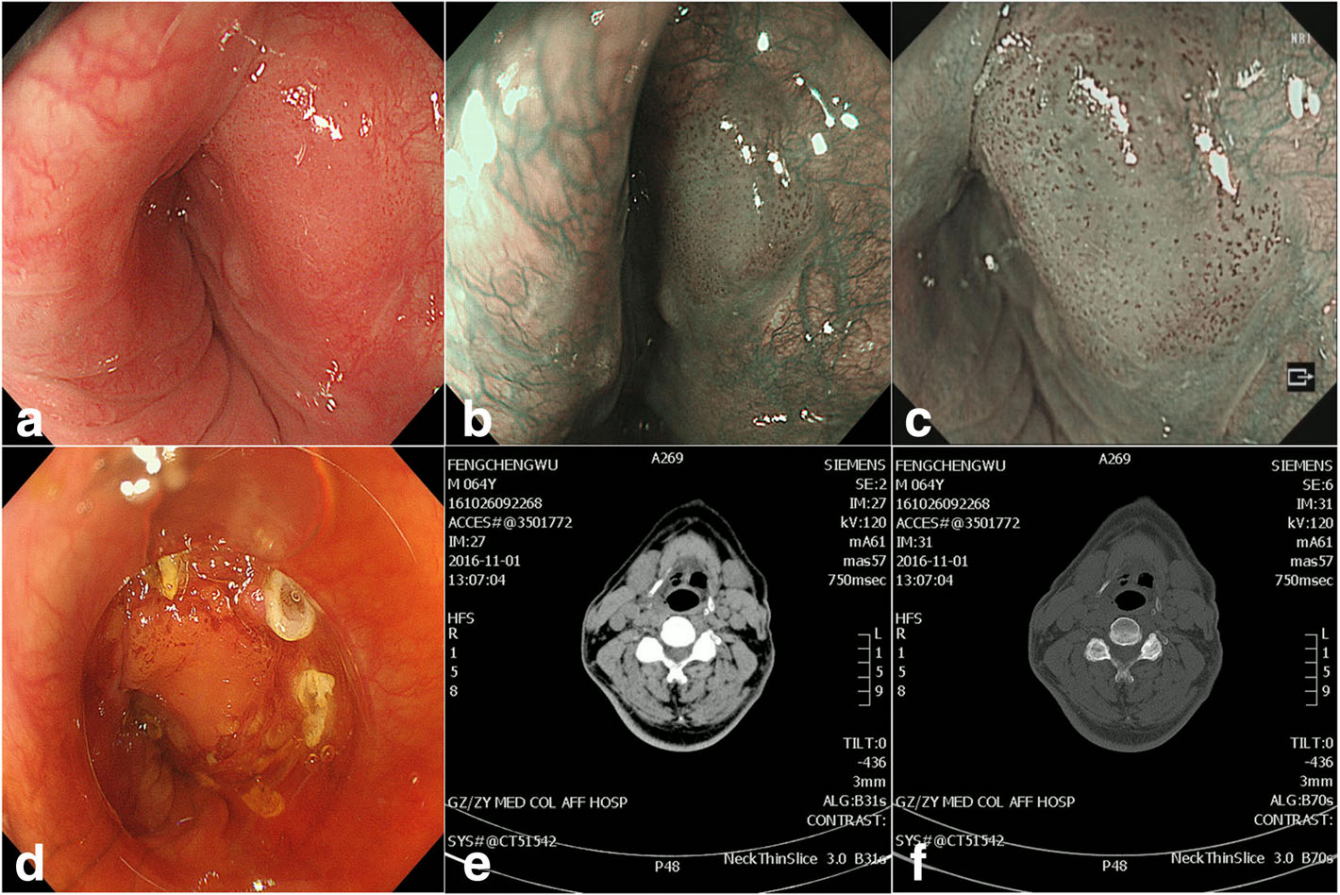
Endoscopic features of superficial pharyngeal cancer in the right pyriform sinus. **a**, Superficial pharyngeal cancer in the right pyriform sinus. **b**, Narrow-band imaging (NBI) showing the pharynx with a well-demarcated brownish area; **c**. Magnified NBI showing an intra-papillary capillary loop type B1 pattern; **d**, The tumour outline was delineated by iodine staining. **e** and **f**. Cervical computed tomographic (CT) view. No lymph node metastasis was identified

A 64-year-old male patient with a history of massive intake of alcohol (40 g/day × 40 years) and tobacco (15/day × 20 years) was admitted to our hospital because of chronic persistent swallowing dysfunction for 2 years. Oesophagogastroduodenoscopy showed a superficial hypopharyngeal cancer in the right pyriform sinus, and we determined the margin and invasion depth of the lesion through white-light endoscopy and 1% iodine, narrow-band imaging (NBI), and magnified NBI (Fig. 1a-d). Cervical computed tomography (CT) showed mild stenosis in the right pyriform sinus and no lymph node metastasis (Fig. 1e and f).

**Fig. 2:**
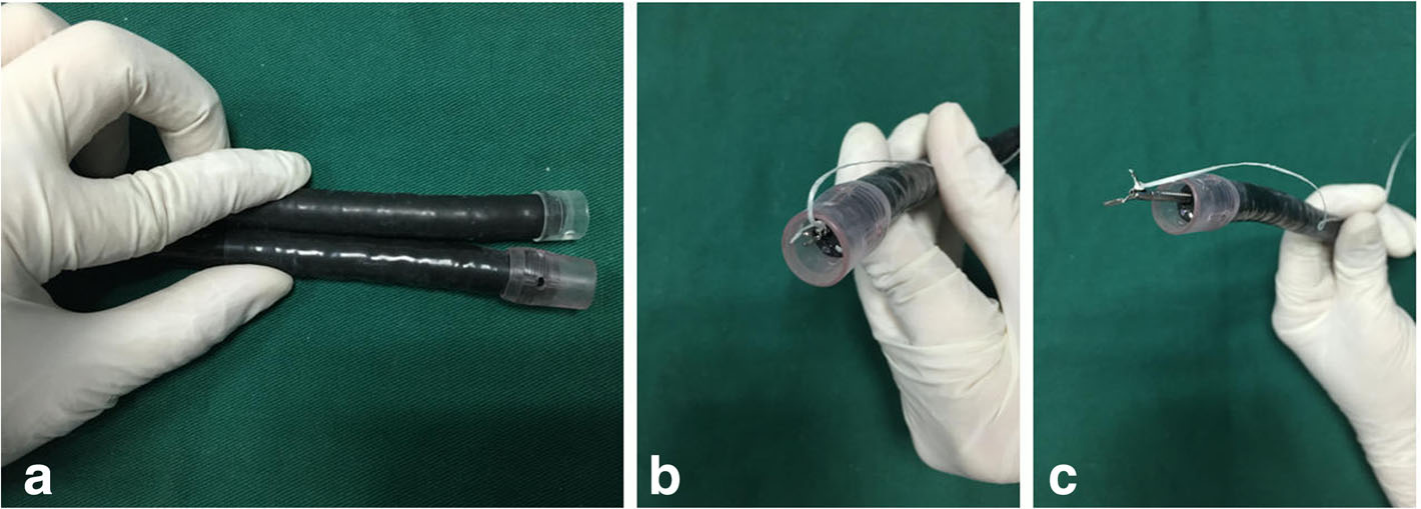
**a** Contrast between two kinds of transparent hood; **b**, A long piece of dental floss is tied to the arm of the haemoclip; **c**, The haemoclip with dental floss is withdrawn into the transparent hood and the accessory channel of the endoscope to enable insertion of the endoscope

**Fig. 3:**
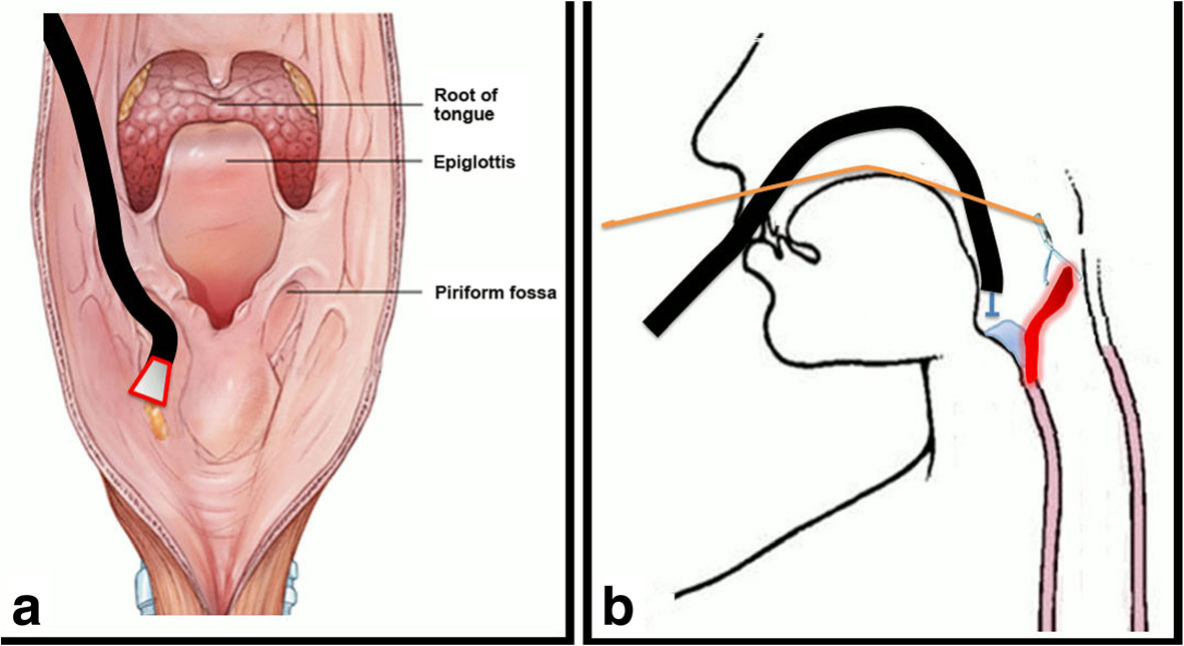
Schema of the procedure. **a**, The transparent hood (Elastic Touch, slit and hole type, M (long), Top company, Tokyo Japan) instead of the laryngoscope is used to create a working space by pushing the larynx; **b**, A haemoclip is placed on the submucosal tissue directly beneath the flap and maintains a clear submucosal dissection plane during endoscopic submucosal dissection

ESD was adopted for the treatment of the lesion. The procedure was performed under anaesthesia by intravenous injection of propofol (AstraZeneca, UK). A H260Z endoscope (Olympus Optical Co, Ltd., Tokyo, Japan) was used. We used a transparent hood (Elastic Touch, slit and hole type, M (long), Top company, Tokyo Japan), longer than a transparent distal hood (D-201-11,804, Olympus) commonly used during ESD, instead of the curved laryngoscope to provide adequate working space by pushing the larynx (Fig. 2a, Fig. 3a). The lesion was first marked with a Dual knife (KD-650Q; Olympus). Then, a solution of indigo carmine and glycerol was injected along the markings to create submucosal lift. The initial incision followed by a circumferential incision was performed using the dual knife. After the circumferential mucosal incision was performed, dental floss was tied to a haemoclip, and the haemoclip was anchored to the subepithelial tissue beneath the mucosal flap to create counter traction and maintain clear visualization of the dissecting plane (Fig. 2b, Fig. 2c, and Fig. 3b). Then, the lesion was resected smoothly. Less than 10 min was needed from placing the haemoclip on the submucosal tissue directly to the final dissection (video for ESD procedure in the Additional file 1, Fig. 4). The lesion was pathologically confirmed as superficial squamous cell carcinoma and resected completely. Detailed pathologic results are shown in Fig. 5. The contrastive analysis for the resected specimen and histopathological examination showed that the lesion was limited in the intraepithelia of pharyngeal mucosa without vascular and neural invasion and the distance of the lesion to closest margin of the resected specimen was 3.01 mm (Fig. 6).

**Fig. 4:**
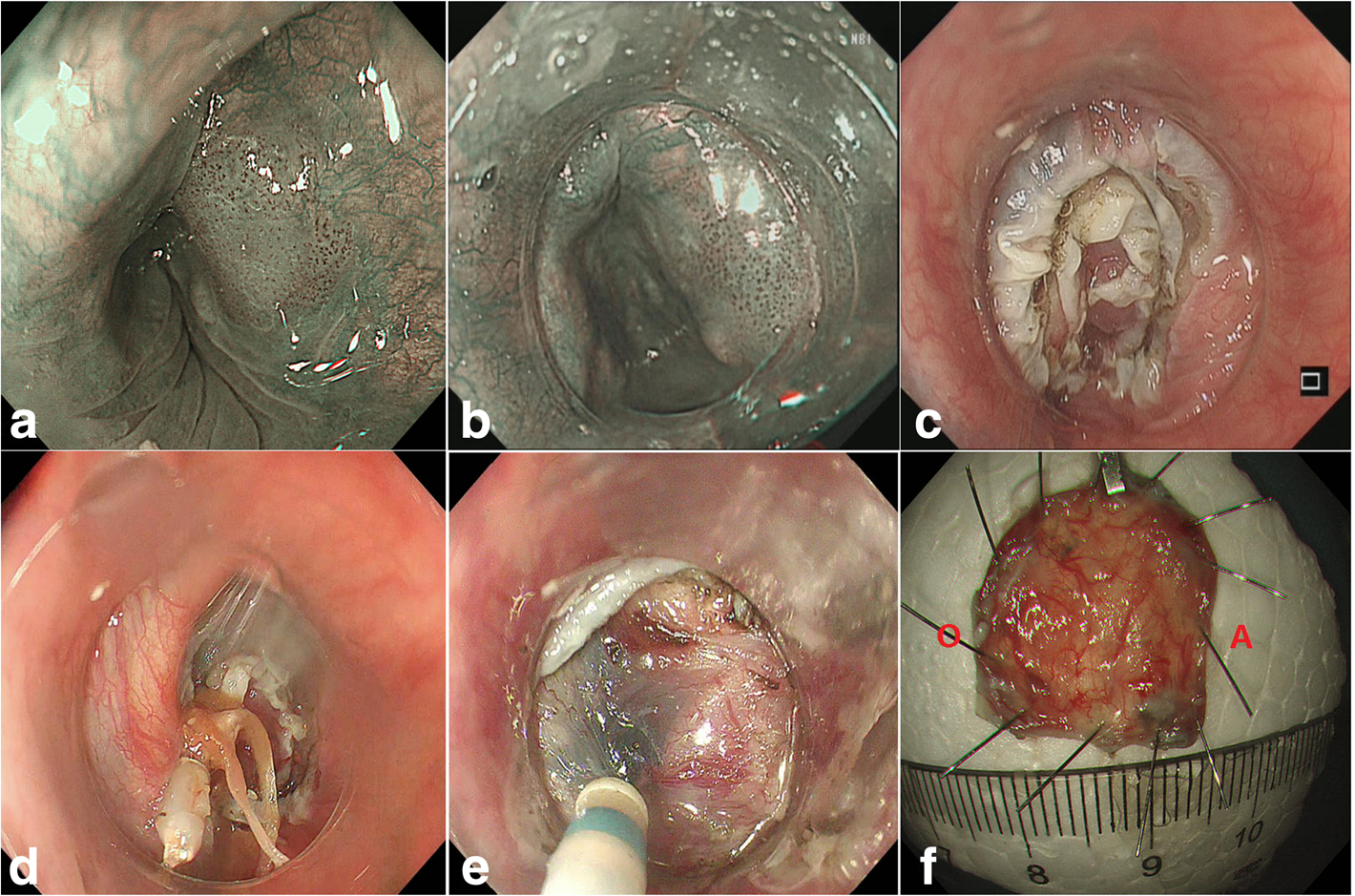
**a** The anal margin of the lesion could not be displayed before using the transparent hood; **b**, The transparent hood could provide a clear view; **c**, A circumferential mucosal incision was performed; **d**, A haemoclip was placed on the submucosal tissue directly beneath the hood and provided proper counter traction during the procedure; **e**, The anchored haemoclip was remarkably helpful for visualizing and dissecting the submucosal tissue during the procedure; **f**, The lesion was resected en bloc and fixed by insect needles. A is anal margin of the resected specimen and O is oral margin of the resected specimen

**Fig. 5:**
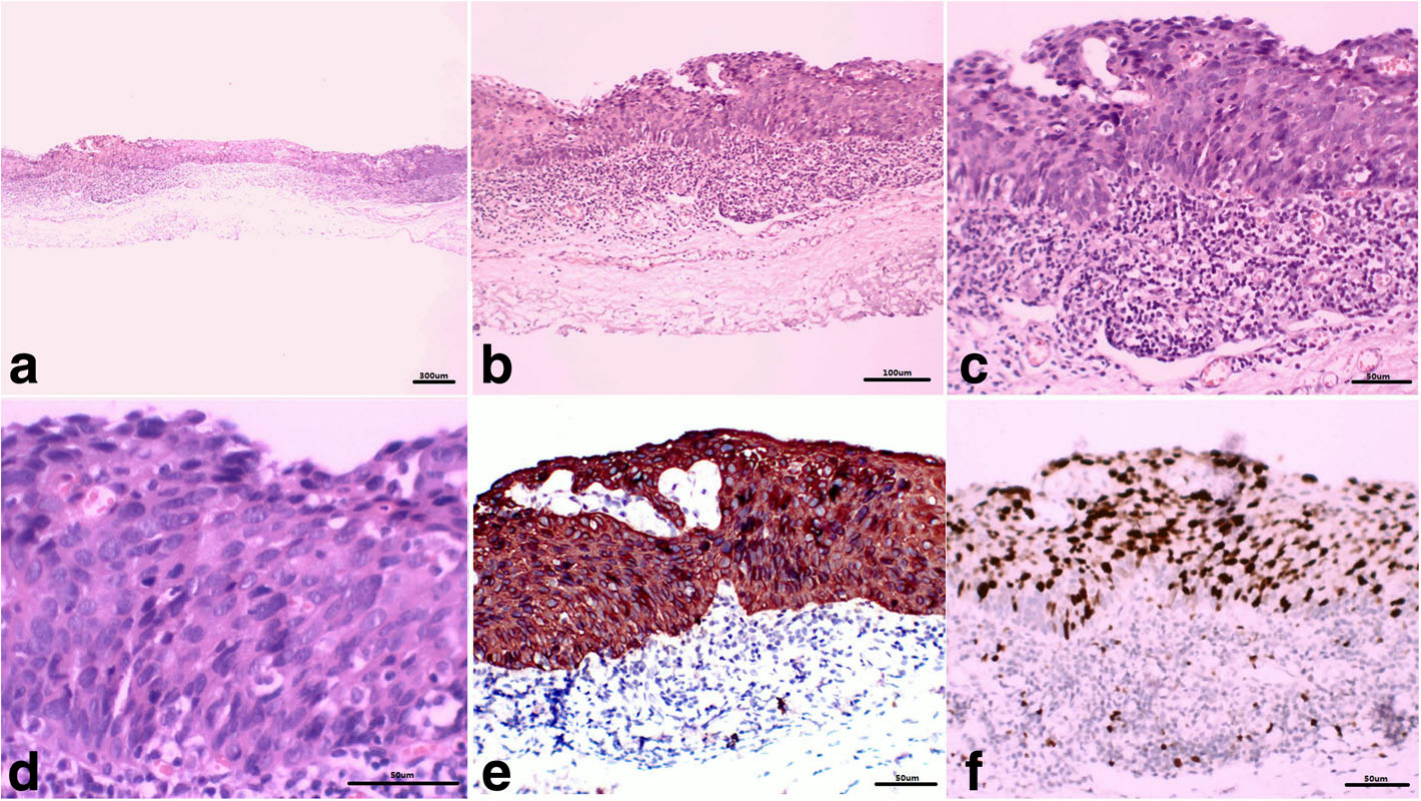
Pathological features of the pharyngeal cancer represented by haematoxylin & eosin (HE) and immunohistochemical staining (IHC). Full-thickness heterotypic cells generated within the epithelial layer and partial basement membrane were broken through (**a**, **b**, **c**, **d**). All the tumour cells were diffusely positive for CK5/6, and the index of Ki-67 was approximately 80% (**e**, **f**)

**Fig. 6:**
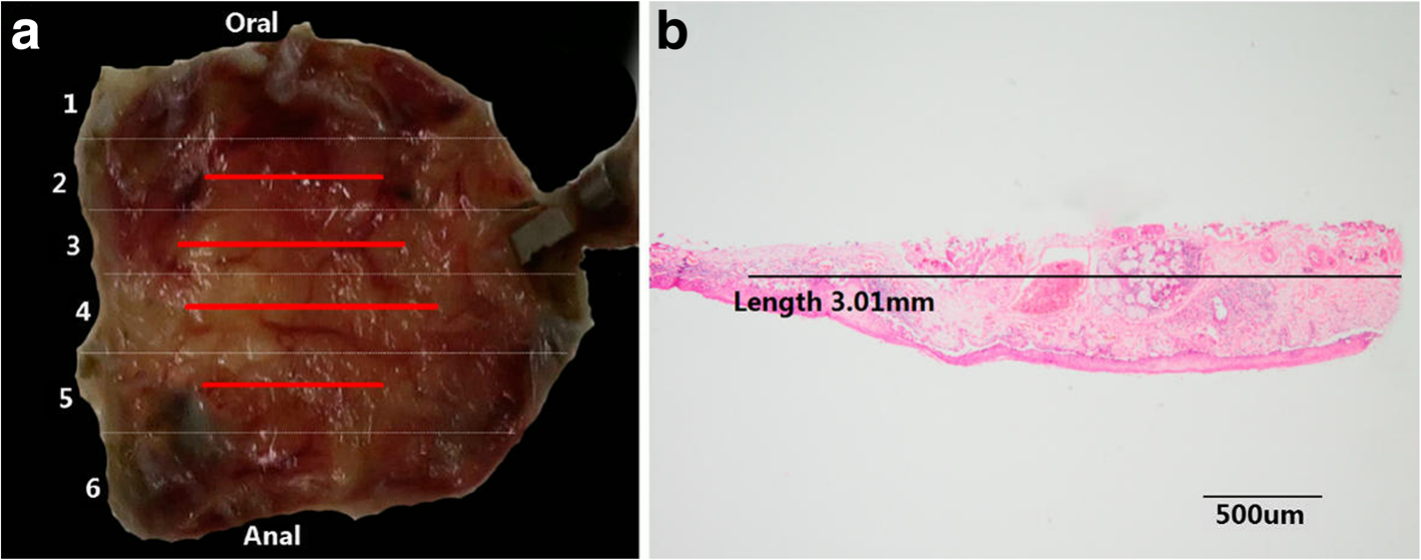
Contrastive analysis for the resected specimen and histopathologic examination. **a**: The resected specimen was cut into slices at each 2 mm width. The red lines represent lesion areas in each slice. Oral is oral margin of the specimen. Anal is anal margin of the specimen. **b**: Histopathologic show for the distance of the lesion to closest margin of the resected specimen

## Discussion and conclusions

It is difficult for gastrointestinal endoscopists to detect early superficial pharyngeal cancer by conventional white light endoscopy because the cancer presents a few morphological changes [4, 5]. However, the introduction of magnifying endoscopy with narrow-band imaging (ME-NBI) allows better detection for superficial pharyngeal lesions [6, 7]. Previously, pharyngeal cancer was usually detected at advanced stages, and its prognosis has been poor [8]. Surgical resection for advanced pharyngeal cancer is necessary, which could cause swallowing disorders, dysgeusia defect, speech problem, and serious cosmetic deformities of the neck [8, 9]. ESD was first developed in the gastrointestinal tract and has been widely used because of its less invasion and good clinical outcomes. The studies have demonstrated that ESD is clinically feasible in the treatment of superficial pharyngeal cancer, with no severe adverse events, and the indications of ESD for superficial pharyngeal cancer are (1) no evidence of invasion to the muscularis mucosa by white-light endoscopy, (2) no lymph node metastasis by cervical ultrasound or computed tomography (CT) examination, and (3) histopathological diagnosis of squamous cell carcinoma [6, 10]. However, ESD of the pharyngeal region is still not well developed because of its narrow and complex space. The success of ESD for superficial hypopharyngeal cancer depends on adequate wide working space and a clear visualization for the dissected lesion. The narrow space of the pharynx makes the endoscope and other devices to interfere with each other. The conventional ESD usually requires an otorhinolaryngologist to create adequate working space by lifting the larynx with a curved laryngoscope, which takes time and increases medical expenses. To overcome these issues, we have designed a novel method, using a transparent hood (Elastic Touch, slit and hole type, M (long), Top company, Tokyo Japan) instead of the laryngoscope to create adequate working space and using dental floss tied to a haemoclip, which is anchored to mucosal tissue, to provide well-visualized dissecting line during ESD of superficial cancer in the hypopharynx region. The traction method has been developed, which makes ESD safer and faster, similar to the clip-with-line method [11, 12]. Iizuka et al. [13] reported the usefulness of endoscopic laryngo–pharyngeal surgery, and during which, Fraenkel laryngeal forceps were used to create proper counter traction to provide well-visualized dissecting line during ESD in the pharyngeal region. However, the disadvantage of the procedure is that the endoscope and other devices still interfere with each other in the narrow space of the pharynx. A major advantage of our new method is that a transparent hood is used to replace the curved laryngoscope to create adequate working space and dental floss tied to a haemoclip is applied for counter traction during ESD so that the devices no longer interfere with each other, which makes ESD in the pharyngeal region feasible and easy.

In conclusion, modified ESD in the hypopharynx region, using a transparent hood to create adequate working space and dental floss tied to a haemoclip to create counter traction, enables early pharyngeal superficial cancer to be removed completely under endoscope by gastroenterologist. This is the first report of modified ESD for a superficial hypopharyngeal cancer.

## Additional file

Additional file 1: A novel method-Lianjun Di video of ESD procedure, this is video of ESD procedure for the patient. (MP4 88400 kb)

## Abbreviations

CT: Computed tomography
ESD: Endoscopic submucosal dissection
ME-NBI: Magnifying endoscopy with narrow-band imaging
NBI: Narrow-band imaging
